# Impact of Colic Pain as a Significant Factor for Predicting the Stone Free Rate of One-Session Shock Wave Lithotripsy for Treating Ureter Stones: A Bayesian Logistic Regression Model Analysis

**DOI:** 10.1371/journal.pone.0123800

**Published:** 2015-04-22

**Authors:** Doo Yong Chung, Kang Su Cho, Dae Hun Lee, Jang Hee Han, Dong Hyuk Kang, Hae Do Jung, Jong Kyou Kown, Won Sik Ham, Young Deuk Choi, Joo Yong Lee

**Affiliations:** 1 Department of Urology, Severance Hospital, Urological Science Institute, Yonsei University College of Medicine, Seoul, Korea; 2 Department of Urology, Gangnam Severance Hospital, Urological Science Institute, Yonsei University College of Medicine, Seoul, Korea; 3 Department of Urology, Severance Check-Up, Yonsei University Health System, Seoul, Korea; 4 Department of Urology, Yangpyeong Health Center, Yangpyeong, Korea; 5 Department of Urology, Haeundae Paik Hospital, Inje University College of Medicine, Busan, Korea; Cedars-Sinai Medical Center, UNITED STATES

## Abstract

**Purpose:**

This study was conducted to evaluate colic pain as a prognostic pretreatment factor that can influence ureter stone clearance and to estimate the probability of stone-free status in shock wave lithotripsy (SWL) patients with a ureter stone.

**Materials and Methods:**

We retrospectively reviewed the medical records of 1,418 patients who underwent their first SWL between 2005 and 2013. Among these patients, 551 had a ureter stone measuring 4–20 mm and were thus eligible for our analyses. The colic pain as the chief complaint was defined as either subjective flank pain during history taking and physical examination. Propensity-scores for established for colic pain was calculated for each patient using multivariate logistic regression based upon the following covariates: age, maximal stone length (MSL), and mean stone density (MSD). Each factor was evaluated as predictor for stone-free status by Bayesian and non-Bayesian logistic regression model.

**Results:**

After propensity-score matching, 217 patients were extracted in each group from the total patient cohort. There were no statistical differences in variables used in propensity- score matching. One-session success and stone-free rate were also higher in the painful group (73.7% and 71.0%, respectively) than in the painless group (63.6% and 60.4%, respectively). In multivariate non-Bayesian and Bayesian logistic regression models, a painful stone, shorter MSL, and lower MSD were significant factors for one-session stone-free status in patients who underwent SWL.

**Conclusions:**

Colic pain in patients with ureter calculi was one of the significant predicting factors including MSL and MSD for one-session stone-free status of SWL.

## Introduction

Since the introduction of shock wave lithotripsy (SWL) in the early 1980s, significant improvements have been made in shockwave technology and safety [1]. For stones in the distal ureter, the stone-free rate for stones <10 mm treated by SWL was 86% compared with a 74% stone-free rate for those >10 mm [2]. Therefore, SWL has become a safe and accepted treatment modality for most intra-renal stones and many ureteral stones [3]. Despite the popular use of SWL, controversies remain regarding its success rate and the number of sessions required achieving stone-free status. Several studies have demonstrated that the consistency, size, shape, location, and Hounsfield units (HU) of the ureteral stone and the BMI of patients have been significant factors that predict the outcome in SWL [4,5].

The success rate or stone-free rate may also be related to other clinical factors including symptom severity, patient expectations, associated infections, solitary kidney, and abnormal ureteral anatomy [6]. Colic pain, as the most common symptom in patients with ureter calculus, was related to ureteral wall edema and hydronephrosis. Ureter stones in patients with colic pain are unlikely to be impacted as they do not have enough time to develop surrounding edema [7,8]. However, in several studies, colic pain as another patient factor has not been evaluated for predicting factors after SWL. Thus, we analyzed patients with ureteral stones who underwent SWL monotherapy. The goals of this study were to evaluate prognostic factors, including colic pain before SWL, to estimate the probability of stone-free status using a Bayesian logistic regression model.

## Materials and Methods

### Patient cohort

Our study had a retrospective nature and the patient records and information were anonymized and deidentified prior to analysis. Medical records were obtained from a database for patients who underwent an initial session of SWL between November 2005 and May 2013 at Severance Hospital, Seoul, Korea. During this period, a total of 1,418 patients were registered in our database. Inclusion criteria for the present study were as follows: 4–20 mm single, radiopaque calculi located within the ureter on non-contrast computed tomography (NCCT), presenting within a month of treatment and without evidence of stone migration. Patients without NCCT scans were excluded. Ultimately, 551 patients, each with a ureter stone, were eligible for our analyses.

### Good clinical practice protocols

The study was carried out in agreement with the applicable laws and regulations, good clinical practices, and ethical principles described in the Declaration of Helsinki. The Institutional Review Board of Severance Hospital approved the study protocol (Approval No. 4-2014-0372).

### Shock wave lithotripsy

From 2005 through 2011, SWL was performed with an electroconductive lithotripter (EDAP Sonolith Praktis, Technomed, Lyon, France). Starting in 2012, SWL was performed with an electromagnetic generative lithotripter (Dornier Compact Delta II lithotripter, Dornier Medtech, Wessling, Germany). All patients were treated under fluoroscopic guidance. The number of shock waves per SWL session varied between 2,500 and 4,000 at a rate of 60 to 90 shock waves per minute. We terminated the session prematurely if the stone became difficult to visualize during the session. The launch intensity was conducted within a focal peak pressure ranging from 16 to 55 MPa, according to the level of pain reported by each patient while SWL was being conducted.

### Demographic data

A detailed history of the ureter stone was obtained, including the number of past stone events, history of pain onset, and stone characteristics. The colic pain as the chief complaint was defined as subjective flank pain during physical examination. Stone characteristics included location, maximal stone length (MSL), mean stone density (MSD), and skin-to-stone distance (SSD). MSD was measured using bone windows on a magnified, axial NCCT image of the stone in the maximal diameter, in which the elliptical region of interest incorporated the largest cross-sectional area of the stone without including adjacent soft tissue. SSD was measured in the axial plane, 45 degrees off the vertical axis. The MSL measured on NCCT was the longest stone length in 3 dimensions. We used the GE Centricity picture archiving and communication system (PACS) during the measurement procedure. SWL treatment of ureter calculi was defined as stone-free status when simple X-ray analysis determined that patients were calcification-free. Patients with stones ≤3mm in diameter without the need for auxiliary measures within a 3-month follow-up period were defined as a treatment success [9]. In addition, no calcification in simple X-ray tithing same follow-up period were defined as a stone-free status.

### Propensity-score matching: Painless versus painful groups

A propensity-score matching study was performed to further elucidate the characteristics of our patients with ureter stones after total cohort analyses. In our study cohort, 288 patients were included in the painful group, which was compared with a matched painless group. Propensity-scores were used to match patients, based on the range of each characteristic [10,11]. Calculations were made for each patient by employing a multivariable logistic regression model using a binomial method based on age, MSL, and MSD, which were noted for patients’ factors on success or stone-free rate. Of the patients who were presenting pain (painful group), those with the most similar propensity scores were selected as the control group (painless group), after optimally matching of 0.5 times the standard deviation of propensity scores. Descriptive statistics characterized the men following propensity-score matching.

### Statistical analysis

Statistical comparisons of continuous variables from patient demographic information were carried out using either Student’s or Welch’s two-sample t-test or the Wilcoxon rank sum test. Categorical variables were compared using Pearson's chi-squared test. Univariate and multivariate non-Bayesian logistic regression with a binomial method were carried out for significant factors of one-session stone-free status. The areas under the curve (AUC) from receiver operator characteristic (ROC) curves for each predictive factor were analyzed using the bootstrap test for two correlated ROC curves. The total scores of the AUCs were interpreted as follows: 0.8–0.9 as an excellent level and 0.7–0.8 as a good level [12]. A Bayesian logistic regression model contains covariates determined by the minimal sufficient adjustment set for the total effects was fitted using second-order penalized quasi-likelihood methods to produce starting values for the second model using Bayesian Markov chain Monte Carlo (MCMC) methods [13]. Consequently, MCMC chains were run for between 50,000 and 300,000 iterations to obtain final posterior parameter distribution of mean value and 95% credible intervals (CI). Optimal cut-off values for significant values were identified from ROC curves using Youden methods. Statistical analyses were performed using R software (version 3.0.3, R Foundation for Statistical Computing, Vienna, Austria; http://www.r-project.org) and its MatchIt package for propensity score matching, its OptimalCutpoints for optimal cut-off value and Zelig and MCMCpack for a Bayesian logistic regression model.

## Results

### Demographic analysis of all patients

The mean age of the patients was 51.76±14.63 years. The distribution of stone location included 449 cases of upper ureter stones (81.5%), 42 cases of mid-ureter stones (7.6%), and 60 cases of lower ureter stones (10.9%). The mean MSL was 9.06±3.93 mm. The MSD was 703.75±267.54 HU and the SSD was 109.72±19.35 mm. The number of patients with pretreatment ureteral stent was 42 (7.6%). The number of cases with one-session stone-free status was 359 (65.1%) (Table 1).

**Table 1:** Demographic data for total patients and for painless versus painful patients.

	Total	Total cohort			Stentless cohort		
		Painless	Painful		Painless	Painful	
	(n = 551)	(n = 263)	(n = 288)	P-value	(n = 232)	(n = 277)	P-value
Sex (M:F)	356:195	176:87	180:108	0.320^a^	155:77	174:103	0.398^a^
Mean age (years) ± SD	51.76±14.63	53.09±14.63	50.55±14.54	0.041^b^	52.76±14.20	50.05±14.48	0.034^b^
Location (%)				0.521^a^			0.530^a^
Upper	449 (81.5)	213 (81.0)	236 (81.9)		187 (80.6)	227 (81.9)	
Mid	42 (7.6)	23 (8.7)	19 (6.6)		21 (9.1)	18 (6.5)	
Lower	60 (10.9)	27 (10.3)	33 (11.5)		24 (10.3)	32 (11.6)	
Maximal stone length (mm)	9.06±3.93	9.61±4.10	8.56±3.69	0.002^b^	9.24±3.69	8.31±3.14	0.002^b^
Mean stone density (HU)	703.75±267.54	744.95±281.94	666.13±248.26	<0.001^b^	742.98±278.88	669.20±249.61	0.002^b^
Skin-to-stone distance (mm)	109.72±19.35	109.70±20.10	109.74±18.68	0.983^b^	110.38±19.51	110.02±18.19	0.262^b^
Stent indwelling (%)	42 (7.6)	31 (11.8)	11 (3.8)	<0.001^a^	0 (0)	0 (0)	-
One-session stone-free (%)	359 (65.1)	152 (57.8)	207 (71.9)	<0.001^a^	138 (59.5)	202 (72.9)	0.002^a^

^a^Pearson's Chi-squared test with Yates' continuity correction

^b^Student's or Welch's two sample t-test

BMI, body mass index; F, female; HU, Hounsfield units; IQR, interquartile range; M, male; SD, standard deviation.

### Painless versus painful groups in total cohort

Among the total patients, 263 patients had no pain and 288 patients had pain before SWL was performed. The mean ages were 53.09±14.63 years in the painless group and 50.55±14.54 years in the painful group (P = 0.041). When evaluating the locations of the treated stones, no statistically significant difference was found. The MSL in the painless group (9.61±4.10 mm) was longer than that in the painful group (8.56±3.69 mm; P = 0.002). The MSD was 744.95±281.94 HU in the painless group and 666.13±248.26 HU in the painful group (P<0.001). In terms of SSD, there was no significant difference between the two groups (painless: 109.70±20.10 mm; painful: 9.74±18.68 mm; P = 0.983). Patients with ureteral stents were 31 (11.8%) in painless group and 11 (3.8%) in painful group (P<0.001). One-session success and stone-free rates were higher in the painful group (74.7% and 71.9%, respectively) than in the painless group (60.5% and 57.8%, respectively; P<0.001 for both comparisons) (Table 1).

### Painless versus painful groups in matched cohort

**Fig 1:**
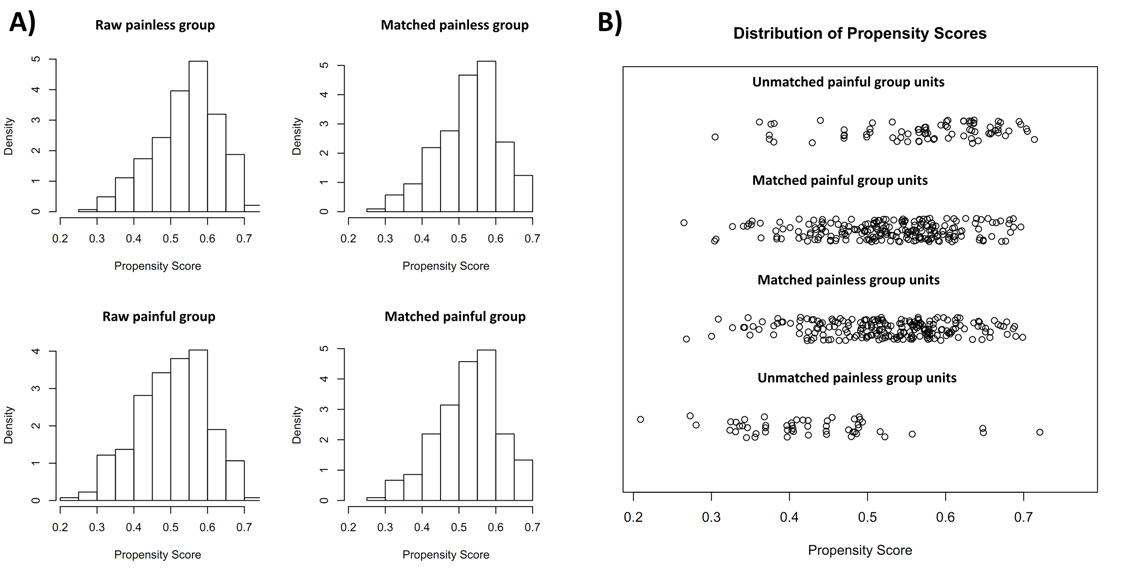
(A) Histogram for propensity-score matching. (B) Jitter plots for propensity-score matching.

Propensity-score matching extracted 217 patients in each group from the total patient cohort. In the painless and painful groups, there were no significant differences in covariables for propensity-scores including age, MSL, and MSD (Fig 1). One-session success and stone-free rate were also higher in the painful group (73.7% and 71.0%, respectively) than in the painless group (63.6% and 60.4%, respectively), following propensity-score matching (Table 2).

**Table 2:** Demographic data for matched patients and for painless versus painful patients in matched cohorts using propensity scores.

	Total	Total matched cohort			Stentless matched cohort		
		Painless	Painful		Painless	Painful	
	(n = 434)	(n = 217)	(n = 217)	P-value	(n = 195)	(n = 208)	P-value
Sex (M:F)	282:152	145:72	137:80	0.481^a^	130:65	133:75	0.639^a^
Mean age (years) ± SD	52.22±14.14	52.24±14.10	52.21±14.21	0.984^b^	52.03±13.27	51.71±14.16	0.819^b^
Location (%)				0.670^a^			0.753^a^
Upper	352 (81.1)	173 (79.7)	179 (82.5)		156 (80.0)	171 (82.2)	
Mid	37 (8.5)	21 (9.7)	16 (7.4)		19 (9.7)	16 (7.7)	
Lower	45 (10.4)	23 (10.6)	22 (10.1)		20 (10.3)	21 (10.1)	
Maximal stone length (mm)	9.04±3.73	9.35±4.05	8.73±3.35	0.084^b^	9.03±3.63	8.53±3.01	0.134^b^
Mean stone density (HU)	698.97±241.26	695.58±242.20	702.36±240.83	0.770^b^	702.74±242.28	706.13±241.46	0.889^b^
Skin-to-stone distance (mm)	10.953±19.31	109.90±20.26	109.17±18.36	0.696^b^	110.91±19.31	109.87±17.94	0.572^b^
Stent indwelling (%)	31 (7.1)	22 (10.1)	9 (4.1)	0.030^a^	0 (0)	0 (0)	-
One-session stone-free (%)	285 (65.7)	131 (60.4)	154 (71.0)	0.026^a^	119 (61.0)	149 (71.6)	0.032^a^

^a^Pearson's Chi-squared test with Yates' continuity correction

^b^Student's or Welch's two sample t-test

BMI, body mass index; F, female; HU, Hounsfield units; IQR, interquartile range; M, male; SD, standard deviation.

### Significant factors in SWL one-session stone-free status

The univariate logistic regression models evaluated a total of 434 patients and revealed that a painful stone (odds ratio [OR]: 1.605; 95% confidential interval [CI]: 1.077–2.400; P = 0.020), shorter MSL (OR: 0.844; 95% CI: 0.793–0.895; P<0.001), and lower MSD (OR: 0.997; 95% CI: 0.996–0.998; P<0.001) were statistically significant factors associated with stone-free status. In multivariate analysis, a painful stone (OR: 1.628; 95% CI: 1.053–2.532; P = 0.029), shorter MSL (OR: 0.872; 95% CI: 0.819–0.925; P<0.001), and lower MSD (OR: 0.997; 95% CI: 0.996–0.998; P<0.001) were independent significant factors for stone-free status in patients who underwent SWL (Table 3). Using a Bayesian logistic regression model, posterior distribution of each factor demonstrate that a painful stone (mean: 0.480; 95% CI: 0.002–0.889), shorter MSL (mean: -0.156; 95% CI: -0.227–-0.094), and lower MSD (mean: -0.003; 95% CI: -0.004–-0.002) were also significant factors for stone-free status (Table 4).

**Table 3:** Univariate and multivariate logistic regression models on one-session stone-free rates in matched patients.

	OR	95% CI	P-value
*Univariate*			
Age (year)	0.994	0.980–1.008	0.387
Sex (male)	0.827	0.541–1.255	0.375
Stone location			
Upper	Reference		
Mid	0.600	0.309–1.180	0.132
Lower	0.771	0.436–1.395	0.380
Pain	1.605	1.077–2.400	0.020
Maximal stone length (mm)	0.844	0.793–0.895	<0.001
Mean stone density (HU)	0.997	0.996–0.998	<0.001
Skin-to-stone distance (cm)	1.003	0.993–1.013	0.581
*Multivariate*			
Pain	1.628	1.053–2.532	0.029
Maximal stone length (mm)	0.872	0.819–0.925	<0.001
Mean stone density (HU)	0.997	0.996–0.998	<0.001

**Table 4:** Posterior distribution for Bayesian logistic regression models on predicting factors of one-session stone-free rates in matched patients with ureter stones.

Parameters	Mean	SD	CI 2.5%	Median	CI 97.5%
Age (year)	-0.009	0.008	-0.027	-0.009	0.007
Sex (Male)	-0.195	0.271	-0.732	-0.185	0.320
Stone location					
Upper	-	-	-	-	-
Mid	-0.026	0.429	-0.930	-0.036	0.787
Lower	-1.128	0.368	-1.874	-1.123	-0.445
Pain	0.480	0.233	0.002	0.489	0.889
Maximal stone length (mm)	-0.156	0.034	-0.227	-0.156	-0.094
Mean stone density (HU)	-0.003	0.001	-0.004	-0.003	-0.002
Skin-to-stone distance (cm)	0.000	0.006	-0.012	0.000	0.012

### ROC curves and cut-off value of MSL and MSD

**Fig 2:**
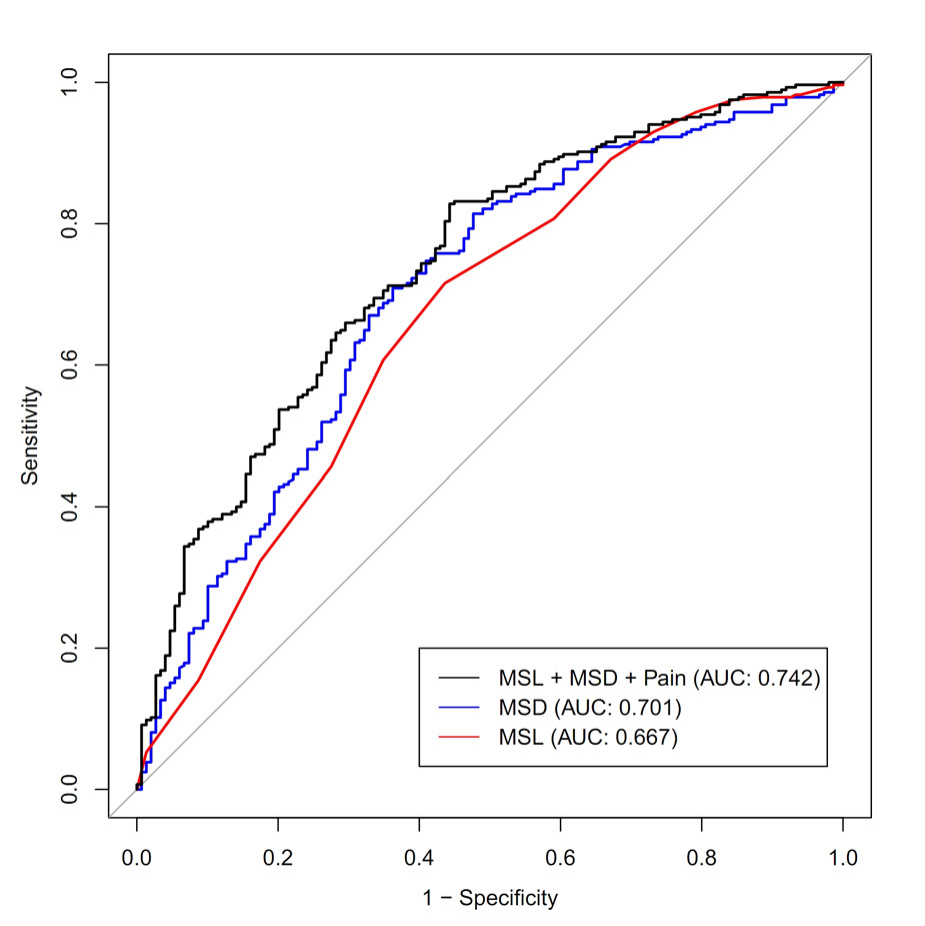
Receiver operator characteristic (ROC) curve and the area under the ROC curve (AUC) of stone-free status following shock wave lithotripsy. The AUC of the three predictive values shown to be significant in multivariate logistic regression analysis was 0.742 (95% CI 0.693–0.790), which was higher than the AUCs for MSL and MSD.

**Fig 3:**
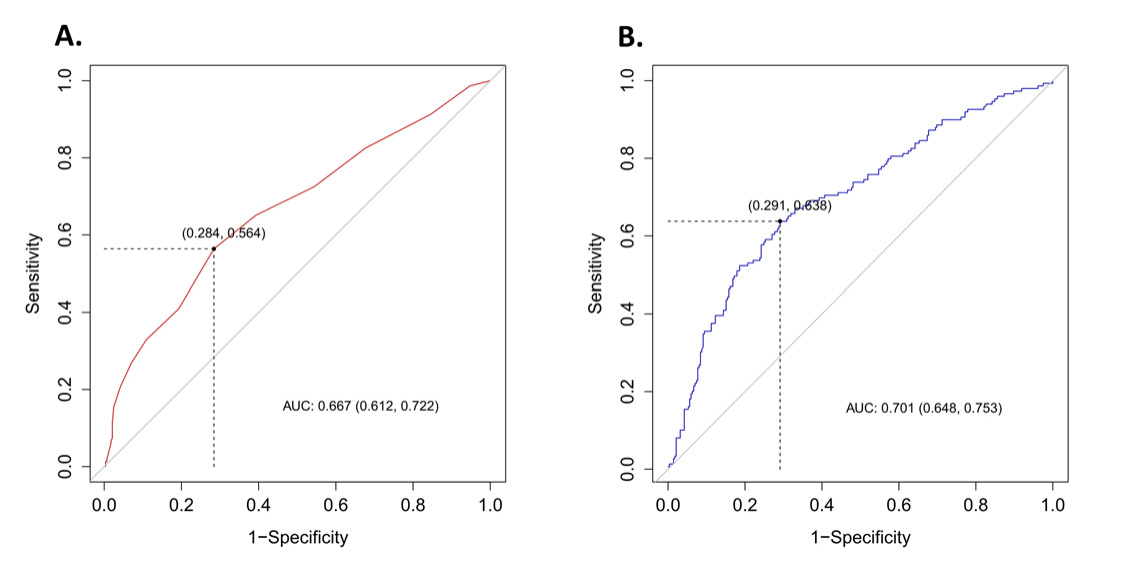
Receiver Operator Characteristic (ROC) curve of stone-free status following shock wave lithotripsy. (A) The area under the ROC curve (AUC) of the MSL was 0.667 (95% CI 0.612–0.722) and the cut-off value was 10.0 mm. (B) The AUC of MSD was 0.701 (95% CI 0.648–0.753) and the cut-off value was 772.6 HU.

For stone-free status, the AUC for MSL was 0.667 (95% CI 0.612–0.722), while that for MSD was 0.701 (95% CI 0.648–0.753). The AUC for the three factors shown to be significant for predicting stone-free status in the multivariate logistic regression analysis was 0.742 (95% CI 0.693–0.790), which was significantly higher than the AUCs for MSL (P<0.001) and MSD (P = 0.043) (Fig 2). The cut-off value of MSL and MSD were 10.0 mm and 772.6 HU (Fig 3). In Table 5, the number of patients with stone-free status were higher in painful group than painless group for MSL≤10 mm (P = 0.019) and MSD≤772.6 HU (P = 0.012).

**Table 5:** Comparison of stone-free status in painless and painful group according to optimal cut-off value for MSD and MSL.

		Painless group	Painful group	P-value^a^
MSD > 772.6 HU	Total No.	68	75	
	No. of stone-free (%)	39	36	0.342
MSD ≤ 772.6 HU	Total No.	149	142	
	No. of stone-free (%)	104	118	0.012
MSL > 10 mm	Total No.	61	55	
	No. of stone-free (%)	28	27	0.875
MSL ≤ 10 mm	Total No.	156	162	
	No. of stone-free (%)	103	127	0.019

^a.^Pearson's Chi-squared test with Yates' continuity correction

MSD: Mean stone density

MSL: Maximal stone length.

## Discussion

In our study, colic pain, MSL, and MSD were significant factors in predicting the one-session stone-free status after SWL. After propensity-score matching, pretreatment factors including sex, age, stone location, MSL, MSD, and SSD demonstrated no statistical differences between painless and painful groups. The rate of stent indwelling was higher in the painless group (10.1%) than in the painful group (4.1%); however, it has been known that routine use of ureteral stents before SWL does not improve stone-free rate [14]. On the contrary, colic pain may be a potential predictor for SWL success. Colic pain, which is one of most common symptom of a urinary stone disease, can have an influence on the success rate for SWL. The cause of colic pain in patients with ureter calculi can be an increase in the intrarenal and intraureteral pressure. The ureteral smooth muscle is very small and uses an active calcium pump to provide contractions, and stone retention may be potentially caused by ureteral spasm, which can increase the intraureteral and intrarenal pressure [15]. The edema of ureteral wall may be also induced by the mechanical irritation from the stone [16]. It has been assumed that ureteral wall edema formation caused over time by a symptomatic impacted ureter stone impairs stone-free status after SWL [17]. Such impacted stones were correlated with the development of mucosal edema within 24–48 hours. It has been observed in histopathologic evaluations that the mucosa, which the stone is in contact with, gains a hyperplastic appearance within 48 hours due to the increased mitosis. The presence of colic pain seemed to be associated with hydronephrosis and a slightly delayed excretion of urine, possibly caused by the beginning of ureteral edema [9]. Obstructing impacted ureter stones may stay in the same position for a certain period of time and if not treated on time, the function of the affected kidney may face a progressive deterioration [18,19]. Therefore, ureteral stones in patients with colic pain, which are much less likely than in non-colic patients to be impacted stones, may be fragmented and passaged after treatment of SWL. Seitz et al. reported that clinically significant changes occur only after 24 hours, with gradual impaction-impairing stone clearance [9]. Their results may be in agreement with the scientific evidence that mucosal edema develops within days after the appearance of impacted stones in the ureter [17].

The MSL or stone area has been a most important predictor for success in patients underwent SWL. Recently, MSD and SSD have been found to be other important predictors for SWL success. MSD, the attenuation value in HUs of calculi on computed tomography, has also been studied to determine its ability to predict the stone-free rate after SWL [20]. The relationship between stone composition and MSD has been also determined by *in vitro* studies [21]. Stones were classified on the basis of urate, phosphate, and oxalate. These classes (urate, Ca, phosphate, oxalate, H, N, C, Mg) were studied, and different HU values were found (urate: 513±197 HU; phosphate: 1660±292 HU; oxalate: 1684±290 HU). Thus, MSD can be a significant predicting factor for SWL success, as it was correlated with stone composition. SSD as a predictor of SWL success remains controversial. Until recently, SSD was a significant factor in a half of published studies, and in the rest studies, there was no significant difference in SSD for success or stone-free rate after SWL [22–27]. In the present study, SSD was also not a significant predictor for one-session stone-free status after SWL. Thus, in our study, cut-off values in each significant factor including MSL and MSD were extracted and they were 10.0 mm in MSL and 772.6 HU in MSD. In painful group, patients with stone which was less than 10.0 mm and had lower than 772.6 HU demonstrated the higher stone-free status than others. In recent reported study, Park et al. compared patient-reported outcomes and stone-free rate after SWL and ureteroscopic stone surgery [28]. In stone-free rate, ureteroscopic surgery was better than SWL, however, patient-reported outcomes of the SWL group was better than ureteroscopic group for voiding symptom and time to return to routine activity. Therefore, in patients with colic pain, shorter MSL and lower MSD, SWL can be recommended for satisfaction of patient-reported outcomes as well as stone-free status.

In the current study, there were some limitations. First, this study was retrospective in nature; it did, however, include a relatively large cohort for ureter stone disease. To overcome the retrospective nature, a Bayesian logistic regression model was performed to reduce bias from standard non-Bayesian approaches. The Bayesian approach is ideally suited to adapting information that accrues during a trial, potentially allowing for smaller more informative trials and for patients to receive better treatment [29]. Nevertheless, non-Bayesian statistics was also applied in our study. This statistical double-checking should make our study more evidence-based and reliable. Second, stone location can serve as a predictive value for success following SWL; however, in our study, location was not a predictor following SWL. The reason why stone location was not significant may relate to the large number of upper ureter stones (> 80%) among the total stones. Third, the low AUCs for each factor, including MSL and MSD, could be another limitation of our study. However, ROCs and AUCs were analyzed to discover predictive values and significant cut-offs for treatment outcomes, not diagnostic accuracy. Additionally, combination of the three significant factors demonstrated a higher AUC than that for each factor individually, and this result can be the basis for a predictive nomogram using a larger cohort. Fourth, in total patients, both painless and painful groups demonstrated different demographic features including age, MSL, MSD, and the rate of pretreatment stent indwelling. Such heterogeneity between the two groups may be the result of selection and informational bias. Hence, propensity-score matching was performed to form better comparator groups [30]; this is a balancing score, wherein the conditional distribution of the pretreatment characteristics, given the propensity score, is the same for the case and control groups [31]. Propensity scores are most commonly estimated in observational studies for patient and background characteristics using a multivariate logistic regression model [32].

## Conclusions

Some influential factors have been described for the passage of fragmented ureter stones after SWL, including hydrostatic pressure proximal to the calculus, edema, inflammation, impacted status, and spasms of the ureter at the site of the stone. Colic pain, which can reflect ureteral wall edema and impacted status, was also one of several significant predictive factors including MSL and MSD for SWL one-session stone-free status.
